# Supplementary figures and images for: Binary cutpoint and the combined effect of systolic and diastolic blood pressure on cardiovascular disease mortality: A community-based cohort study
Source: PLoS One. 2022 Jun 30;17(6):e0270510. doi: 10.1371/journal.pone.0270510 (PMC9246156; doi:10.1371/journal.pone.0270510)

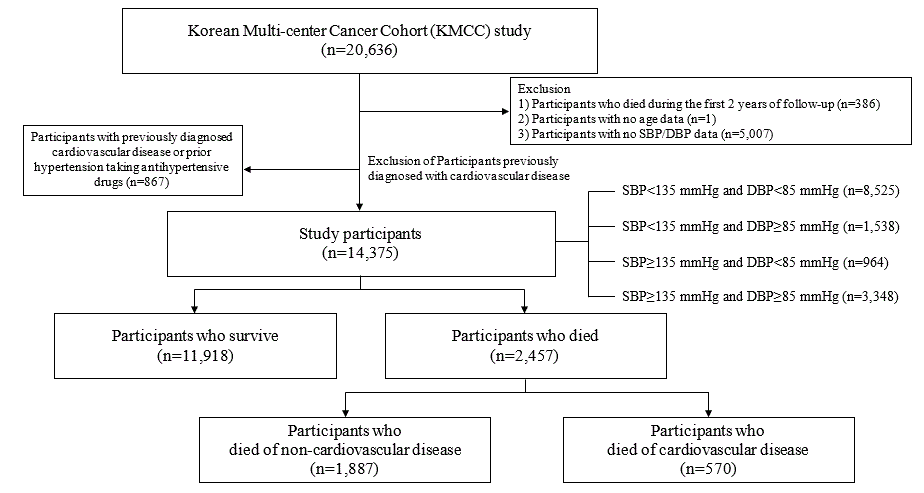

Supplement: S1 Fig — Overview of the study participants from the KMCC study used to analyze the association between BP and cardiovascular death (Abbreviation: SBP, systolic blood pressure; DBP, diastolic blood pressure). (TIF) [file pone.0270510.s001.tif]
