# Supplementary material for: Binary cutpoint and the combined effect of systolic and diastolic blood pressure on cardiovascular disease mortality: A community-based cohort study
Source: PLoS One. 2022 Jun 30;17(6):e0270510. doi: 10.1371/journal.pone.0270510 (PMC9246156; doi:10.1371/journal.pone.0270510)
Supplement: S1 Table — (DOCX) [file pone.0270510.s002.docx]

**Supplementary Table 1.** Classification of the cause of death based on the ICD-10

| Cause of death | ICD-10 code |
| --- | --- |
| All-cause | A00-Z99 |
| Cardiovascular diseases | I00-I99 |
| Ischemic heart diseases | I20-I25 |
| Acute myocardial infarction | I21 |
| Stroke | I60-I69 |
| Cerebral hemorrhage | I60-I62 |
| Cerebral infarction | I63 |
| Other strokes | I65-I69 |
| Hypertensive disease | I10-I16 |
| All-cancer | C00-C97 |
| Non-disease | S00-T98 or V09-Y98 |
| Injury, poisoning and certain other consequence of external causes | S00-T98 |
| External causes of morbidity and mortality | Y09-Y98 |
