# Supplementary material for: Binary cutpoint and the combined effect of systolic and diastolic blood pressure on cardiovascular disease mortality: A community-based cohort study
Source: PLoS One. 2022 Jun 30;17(6):e0270510. doi: 10.1371/journal.pone.0270510 (PMC9246156; doi:10.1371/journal.pone.0270510)
Supplement: S2 Table — (DOCX) [file pone.0270510.s003.docx]

**Supplementary Table 2**. General characteristics of study participants by systolic blood pressure and diastolic blood pressure of 14,375 cohort participants in the Korean Multi-center Cancer Cohort study (KMCC) over 15 follow-up years

|  | Systolic blood pressure (mmHg) ^1^ | | | |  | Diastolic blood pressure (mmHg) ^1^ | | | |
| --- | --- | --- | --- | --- | --- | --- | --- | --- | --- |
|  | **<120** | **120-129** | **130-139** | **140+** |  | **<75** | **75-84** | **85-94** | **95+** |
|  | **Mean (SD)** | **Mean (SD)** | **Mean (SD)** | **Mean (SD)** |  | **Mean (SD)** | **Mean (SD)** | **Mean (SD)** | **Mean (SD)** |
| Age (years) | 48.9 (15.8) | 50.7 (15.4) | 53.5 (14.5) | 59.1 (12.4) |  | 50.4 (16.6) | 52.9 (15.0) | 54.2 (13.8) | 58.8 (11.3) |
| BMI (kg/m^2^) | 22.6 (3.1) | 23.5 (3.2) | 23.9 (3.2) | 24.4 (3.3) |  | 22.7 (3.1) | 23.6 (3.2) | 24.3 (3.3) | 24.7 (3.4) |
| WHR | 0.88 (0.07) | 0.88 (0.07) | 0.89 (0.07) | 0.90 (0.06) |  | 0.88 (0.07) | 0.89 (0.07) | 0.90 (0.06) | 0.90 (0.06) |
| Total cholesterol (mg/dl) | 183.6 (41.1) | 189.4 (40.4) | 194.4 (42.0) | 200.9 (42.4) |  | 184.5 (40.8) | 191.5 (41.4) | 196.4 (42.3) | 205.6 (42.3) |
| Triglyceride (mg/dl) | 129.4 (95.0) | 140.2 (102.5) | 158.7 (116.6) | 177.2 (129.5) |  | 136.1 (102.2) | 149.5 (109.1) | 161.1 (121.9) | 185.0 (131.5) |
| LDL (mg/dl) | 115.6 (37.8) | 115.4 (35.0) | 118.5 (38.2) | 121.6 (40.0) |  | 116.8 (37.0) | 116.4 (37.0) | 119.4 (38.8) | 122.7 (41.3) |
| HDL^1^ (mg/dl) | 50.4 (13.0) | 49.7 (13.3) | 49.7 (12.6) | 49.5 (14.3) |  | 50.1 (13.5) | 49.5 (12.9) | 50.1 (14.0) | 49.6 (13.5) |
| Fasting glucose (mg/dl) | 91.1 (32.4) | 93.2 (33.0) | 95.5 (33.9) | 100.6 (39.6) |  | 92.9 (33.6) | 93.8 (32.9) | 98.0 (38.6) | 99.2 (38.1) |
|  |  |  |  |  |  |  |  |  |  |
|  | **N (%)** | **N (%)** | **N (%)** | **N (%)** |  | **N (%)** | **N (%)** | **N (%)** | **N (%)** |
| Male | 1,390 (33.4) | 1,334 (40.1) | 1,313 (44.4) | 1,675 (42.7) |  | 1,544 (34.2) | 1,981 (39.9) | 1,519 (46.4) | 668 (41.4) |
| DM ^2^ | 304 (7.3) | 295 (8.9) | 331 (11.2) | 594 (15.1) |  | 383 (8.5) | 485 (9.8) | 425 (13.0) | 231 (14.3) |
| Anemia ^2^ | 970 (23.3) | 662 (19.9) | 485 (16.4) | 609 (15.5) |  | 1,139 (25.2) | 913 (18.4) | 456 (13.9) | 218 (13.5) |
| Family history of |  |  |  |  |  |  |  |  |  |
| CVD | 275 (6.6) | 274 (8.3) | 281 (9.5) | 428 (10.9) |  | 283 (6.3) | 433 (8.7) | 336 (10.3) | 206 (12.8) |
| Hypertension | 177 (4.3) | 189 (5.7) | 193 (6.5) | 338 (8.6) |  | 183 (4.1) | 290 (5.8) | 255 (7.8) | 169 (10.5) |
| DM | 169 (4.1) | 185 (5.6) | 121 (4.1) | 212 (5.4) |  | 196 (4.3) | 243 (4.9) | 143 (4.4) | 105 (6.5) |
| Current smoking ^2^ | 1,014 (24.3) | 878 (26.4) | 782 (26.4) | 1,011 (25.8) |  | 1,083 (24.0) | 1,308 (26.3) | 889 (27.2) | 405 (25.1) |
| Current alcohol drinking | 1,410 (33.8) | 1,255 (37.8) | 1,195 (40.4) | 1,582 (40.3) |  | 1,480 (32.7) | 1,908 (38.4) | 1,391 (42.5) | 663 (41.1) |
| Low physical activity^2^ | 1,925 (46.2) | 1,621 (48.8) | 1,440 (48.7) | 2,043 (52.1) |  | 2,126 (47.0) | 2,451 (49.3) | 1,633 (49.9) | 819 (50.8) |

Abbreviation: BMI, Body mass index; WHR, Waist to hip ratio; LDL, low density lipoprotein-cholesterol; HDL, high density lipoprotein-cholesterol; DM, Diabetes mellitus; CVD, Cardiovascular diseases

1. To test the difference of variables between the four groups according to the BP level, an ANOVA was used for continuous variables and a chi-square test was used for count variables. Differences of continuous variables among different SBP and DBP groups were compared with the use of an ANOVA test. Most of the variables, except for HDL cholesterol levels, were statistically significant for each group according to the BP level.
2. ‘DM’ was defined as ‘the subjects who was diagnosed with diabetes based on past history and used antidiabetic drugs’ or ‘those having an FBS ≥ 126 mg/dL at baseline). Anemia was defined as ‘'Hemoglobin levels ≤ 13 mg/dL for men and ≤ 12 mg/dL for women’. Current smokers were defined as those with at least 400 smokers. Low physical activity meant ‘None to ≤ 2 time/week’
