# Supplementary material for: Binary cutpoint and the combined effect of systolic and diastolic blood pressure on cardiovascular disease mortality: A community-based cohort study
Source: PLoS One. 2022 Jun 30;17(6):e0270510. doi: 10.1371/journal.pone.0270510 (PMC9246156; doi:10.1371/journal.pone.0270510)
Supplement: S3 Table — (DOCX) [file pone.0270510.s004.docx]

**Supplementary Table 3.** The association with systolic blood pressure on different specific categories for the risk of mortality in the Korean Multi-center Cancer Cohort study over 15 follow-up years

|  | Death | HR (95% CI) ^1^ | Death | HR (95% CI) ^1^ | Death | HR (95% CI) ^1^ | Death | HR (95% CI) ^1^ | P-trend |
| --- | --- | --- | --- | --- | --- | --- | --- | --- | --- |
|  |  | SBP < 120 | 120 ≤ SBP < 140 | | 140 ≤ SBP < 150 | | 150 ≤ SBP | |  |
| All-cause | 554 | 1.00 | 964 | 1.02 (0.90-1.16) | 354 | 1.12 (1.01-1.28) | 585 | 1.29 (1.08-1.51) | <0.001 |
| CVD | 87 | 1.00 | 220 | 1.30 (0.97-1.73) | 86 | 1.42 (1.00-2.04) | 177 | 1.72 (1.19-2.47) | <0.001 |
| IHD | 27 | 1.00 | 62 | 0.94 (0.54-1.63) | 18 | 0.71 (0.34-1.47) | 43 | 0.97 (0.48-1.98) | 0.89 |
| AMI | 19 | 1.00 | 38 | 0.76 (0.39-1.48) | 11 | 0.55 (0.22-1.37) | 19 | 0.54 (0.21-1.37) | 0.17 |
| Stroke | 38 | 1.00 | 91 | 1.37 (0.89-2.11) | 47 | 2.03 (1.20-3.42) | 92 | 2.36 (1.39-4.01) | <0.001 |
| Hemorrhagic stroke | 16 | 1.00 | 30 | 0.88 (0.42-1.83) | 6 | 0.51 (0.17-1.54) | 28 | 1.53 (0.59-3.93) | 0.30 |
| Ischemic stroke | 10 | 1.00 | 26 | 1.76 (0.79-3.89) | 15 | 1.98 (0.99-4.45) | 21 | 2.58 (1.001-6.97) | 0.03 |
| Hypertension | 6 | 1.00 | 24 | 1.34 (0.54-3.36) | 5 | 0.86 (0.24-3.08) | 19 | 1.89 (0.60-6.00) | 0.36 |
| Cancer | 198 | 1.00 | 291 | 0.94 (0.76-1.17) | 118 | 1.20 (0.90-1.61) | 136 | 1.09 (0.79-1.50) | 0.34 |
| Non-disease | 72 | 1.00 | 99 | 0.76 (0.52-1.10) | 28 | 0.67 (0.39-1.16) | 48 | 0.93 (0.54-1.61) | 0.80 |
|  |  | SBP < 120 | 120 ≤ SBP < 135 | | 135 ≤ SBP < 150 | | 150 ≤ SBP | |  |
| All-cause | 554 | 1.00 | 891 | 1.01 (0.89-1.15) | 427 | 1.13 (1.01-1.31) | 585 | 1.30 (1.12-1.52) | <0.001 |
| CVD | 87 | 1.00 | 205 | 1.28 (0.96-1.71) | 101 | 1.43 (1.02-2.01) | 177 | 1.90 (1.37-2.64) | <0.001 |
| IHD | 27 | 1.00 | 62 | 0.99 (0.57-1.71) | 18 | 0.62 (0.31-1.25) | 43 | 1.07 (0.57-2.00) | 0.99 |
| AMI | 19 | 1.00 | 38 | 0.82 (0.42-1.59) | 11 | 0.49 (0.21-1.17) | 19 | 0.62 (0.28-1.40) | 0.15 |
| Stroke | 38 | 1.00 | 84 | 1.34 (0.87-2.06) | 54 | 2.02 (1.24-3.29) | 92 | 2.71 (1.67-4.39) | <0.001 |
| Hemorrhagic stroke | 16 | 1.00 | 29 | 0.92 (0.44-1.91) | 7 | 0.53 (0.19-1.47) | 28 | 1.68 (0.73-3.86) | 0.10 |
| Ischemic stroke | 10 | 1.00 | 18 | 1.63 (0.73-3.64) | 23 | 2.58 (1.04-6.43) | 21 | 2.54 (1.02-6.70) | 0.03 |
| Hypertension | 8 | 1.00 | 21 | 1.26 (0.50-3.22) | 8 | 1.05 (0.34-3.27) | 19 | 1.74 (0.60-5.02) | 0.30 |
| Cancer | 198 | 1.00 | 266 | 0.92 (0.74-1.15) | 143 | 1.20 (0.92-1.57) | 136 | 1.07 (0.81-1.42) | 0.20 |
| Non-disease | 72 | 1.00 | 93 | 0.76 (0.52-1.11) | 34 | 0.67 (0.41-1.09) | 48 | 0.88 (0.55-1.43) | 0.76 |
|  |  | SBP < 120 | 120 ≤ SBP < 130 | | 130 ≤ SBP < 140 | | 140 ≤ SBP | |  |
| All-cause | 554 | 1.00 | 456 | 0.97 (0.85-1.11) | 508 | 1.10 (0.98-1.25) | 939 | 1.25 (1.07-1.46) | 0.003 |
| CVD | 87 | 1.00 | 92 | 1.15 (0.84-1.58) | 128 | 1.47 (1.06-2.03) | 263 | 1.71 (1.22-2.39) | <0.001 |
| IHD | 27 | 1.00 | 26 | 0.88 (0.48-1.60) | 36 | 1.00 (0.54-1.87) | 61 | 0.93 (0.48-1.80) | 0.92 |
| AMI | 19 | 1.00 | 17 | 0.77 (0.37-1.58) | 21 | 0.79 (0.37-1.69) | 30 | 0.61 (0.27-1.40) | 0.27 |
| Stroke | 38 | 1.00 | 37 | 1.18 (0.73-1.90) | 54 | 1.59 (1.03-2.60) | 139 | 2.28 (1.39-3.75) | <0.001 |
| Hemorrhagic stroke | 16 | 1.00 | 10 | 0.64 (0.27-1.52) | 20 | 1.14 (0.50-2.63) | 34 | 1.11 (0.45-2.71) | 0.57 |
| Ischemic stroke | 10 | 1.00 | 14 | 1.37 (0.70-3.20) | 20 | 1.57 (0.98-3.30) | 28 | 1.98 (1.001-4.56) | 0.045 |
| Hypertension | 8 | 1.00 | 10 | 1.14 (0.42-3.13) | 14 | 1.50 (0.54-4.17) | 24 | 1.54 (0.53-4.50) | 0.39 |
| Cancer | 198 | 1.00 | 145 | 0.91 (0.72-1.15) | 146 | 1.01 (0.77-1.31) | 254 | 1.17 (0.89-1.55) | 0.19 |
| Non-disease | 72 | 1.00 | 46 | 0.70 (0.46-1.05) | 53 | 0.86 (0.55-1.34) | 76 | 0.86 (0.53-1.41) | 0.75 |

Abbreviation: CVD, Cardiovascular diseases; IHD, Ischemic heart diseases; AMI, Acute myocardial infarction

1. Adjusted for age, sex, past medical history of diabetes mellitus, family history of cardiovascular disease, BMI, cigarette smoking, alcohol consumption, physical activity, level of high-density lipoprotein by using a Cox proportional hazards regression analysis.
