# Supplementary material for: Binary cutpoint and the combined effect of systolic and diastolic blood pressure on cardiovascular disease mortality: A community-based cohort study
Source: PLoS One. 2022 Jun 30;17(6):e0270510. doi: 10.1371/journal.pone.0270510 (PMC9246156; doi:10.1371/journal.pone.0270510)
Supplement: S4 Table — (DOCX) [file pone.0270510.s005.docx]

**Supplementary Table 4.** The association with diastolic blood pressure on different specific categories for the risk of mortality in the Korean Multi-center Cancer Cohort study over 15 follow-up years

|  | Death | HR (95% CI) ^1^ | Death | HR (95% CI) ^1^ | Death | HR (95% CI) ^1^ | Death | HR (95% CI) ^1^ | P-trend |
| --- | --- | --- | --- | --- | --- | --- | --- | --- | --- |
|  | DBP < 80 | | 80 ≤ DBP < 90 | | 90 ≤ DBP < 100 | | 100 ≤ DBP | |  |
| All-cause | 843 | 1.00 | 741 | 0.98 (0.87-1.10) | 525 | 0.99 (0.86-1.14) | 348 | 1.03 (0.87-1.23) | 0.78 |
| CVD | 149 | 1.00 | 174 | 1.17 (0.91-1.51) | 135 | 1.23 (0.92-1.65) | 112 | 1.49 (1.06-2.10) | 0.03 |
| IHD | 38 | 1.00 | 49 | 1.51 (0.91-2.51) | 36 | 1.58 (0.87-2.85) | 27 | 1.87 (0.92-3.81) | 0.12 |
| AMI | 24 | 1.00 | 30 | 1.70 (0.90-3.21) | 20 | 1.85 (0.86-3.96) | 13 | 2.31 (0.88-6.04) | 0.10 |
| Stroke | 69 | 1.00 | 73 | 1.03 (0.71-1.50) | 68 | 1.21 (0.79-1.84) | 58 | 1.43 (0.88-2.34) | 0.15 |
| Hemorrhagic stroke | 21 | 1.00 | 24 | 1.53 (0.76-3.07) | 20 | 1.84 (0.81-4.19) | 15 | 1.79 (0.68-4.73) | 0.22 |
| Ischemic stroke | 22 | 1.00 | 20 | 0.82 (0.42-1.62) | 21 | 1.11 (0.52-2.35) | 9 | 0.65 (0.24-1.77) | 0.65 |
| Hypertension | 14 | 1.00 | 21 | 1.47 (0.67-3.22) | 10 | 0.94 (0.35-2.54) | 11 | 1.32 (0.44-4.02) | 0.85 |
| Cancer | 282 | 1.00 | 224 | 0.89 (0.72-1.09) | 154 | 0.89 (0.69-1.15) | 83 | 0.85 (0.60-1.18) | 0.32 |
| Non-disease | 89 | 1.00 | 80 | 1.13 (0.78-1.63) | 51 | 1.11 (0.70-1.75) | 27 | 1.01 (0.55-1.85) | 0.87 |
|  | DBP < 75 | | 75 ≤ DBP < 85 | | 85 ≤ DBP < 95 | | 95 ≤ DBP | |  |
| All-cause | 706 | 1.00 | 797 | 0.99 (0.89-1.10) | 561 | 0.96 (0.85-1.10) | 393 | 1.05 (0.89-1.25) | 0.82 |
| CVD | 124 | 1.00 | 177 | 1.25 (0.99-1.58) | 141 | 1.33 (1.01-1.76) | 128 | 1.77 (1.27-2.47) | <0.001 |
| IHD | 35 | 1.00 | 49 | 1.27 (0.82-1.98) | 36 | 1.32 (0.77-2.24) | 30 | 1.73 (0.89-3.36) | 0.12 |
| AMI | 22 | 1.00 | 31 | 1.35 (0.78-2.36) | 19 | 1.29 (0.65-2.58) | 15 | 1.96 (0.80-4.78) | 0.19 |
| Stroke | 53 | 1.00 | 73 | 1.20 (0.84-1.73) | 75 | 1.59 (1.05-2.39) | 67 | 2.00 (1.23-3.24) | 0.003 |
| Hemorrhagic stroke | 16 | 1.00 | 25 | 1.32 (0.85-2.06) | 20 | 2.00 (0.98-4.56) | 19 | 2.65 (1.06-6.66) | 0.02 |
| Ischemic stroke | 19 | 1.00 | 16 | 0.72 (0.37-1.43) | 26 | 1.52 (0.75-3.07) | 11 | 0.91 (0.35-2.39) | 0.67 |
| Hypertension | 12 | 1.00 | 21 | 1.46 (0.71-3.03) | 12 | 1.03 (0.41-2.59) | 11 | 1.16 (0.39-3.45) | 0.89 |
| Cancer | 235 | 1.00 | 244 | 0.89 (0.74-1.07) | 171 | 0.85 (0.68-1.08) | 93 | 0.81 (0.59-1.12) | 0.14 |
| Non-disease | 77 | 1.00 | 87 | 0.98 (0.71-1.33) | 51 | 0.81 (0.53-1.22) | 32 | 0.86 (0.49-1.49) | 0.40 |
|  | DBP < 75 | | 75 ≤ DBP < 90 | | 90 ≤ DBP < 95 | | 95 ≤ DBP | |  |
| All-cause | 706 | 1.00 | 878 | 0.97 (0.87-1.08) | 480 | 0.98 (0.85-1.14) | 393 | 1.15 (0.99-1.34) | 0.07 |
| CVD | 124 | 1.00 | 199 | 1.14 (0.89-1.45) | 119 | 1.13 (0.83-1.53) | 128 | 1.66 (1.23-2.25) | 0.008 |
| IHD | 35 | 1.00 | 52 | 1.17 (0.74-1.87) | 33 | 1.26 (0.69-2.28) | 30 | 1.64 (0.89-3.02) | 0.12 |
| AMI | 22 | 1.00 | 32 | 1.24 (0.69-2.23) | 18 | 1.32 (0.61-2.86) | 15 | 1.58 (0.71-3.56) | 0.28 |
| Stroke | 53 | 1.00 | 89 | 1.14 (0.79-1.66) | 59 | 1.26 (0.98-1.78) | 67 | 1.95 (1.26-3.04) | 0.006 |
| Hemorrhagic stroke | 16 | 1.00 | 29 | 1.38 (0.70-2.69) | 16 | 1.31 (0.57-3.04) | 19 | 2.35 (1.01-5.70) | 0.03 |
| Ischemic stroke | 19 | 1.00 | 23 | 0.93 (0.48-1.81) | 19 | 1.34 (0.61-2.97) | 11 | 1.03 (0.42-2.50) | 0.73 |
| Hypertension | 12 | 1.00 | 23 | 1.23 (0.58-2.62) | 10 | 0.82 (0.31-2.17) | 11 | 1.15 (0.44-3.03) | 0.98 |
| Cancer | 235 | 1.00 | 271 | 0.89 (0.74-1.08) | 144 | 0.91 (0.70-1.18) | 93 | 0.89 (0.66-1.18) | 0.44 |
| Non-disease | 77 | 1.00 | 92 | 0.92 (0.66-1.28) | 46 | 0.88 (0.56-1.40) | 32 | 0.97 (0.59-1.61) | 0.86 |

Abbreviation: CVD, Cardiovascular diseases; IHD, Ischemic heart diseases; AMI, Acute myocardial infarction

1. Adjusted for age, sex, past medical history of diabetes mellitus, family history of cardiovascular disease, BMI, cigarette smoking, alcohol consumption, physical activity, level of high-density lipoprotein by using a Cox proportional hazards regression analysis.
